# Cocaine/crack use is not associated with fibrosis progression measured by AST-to-Platelet Ratio Index in HIV-HCV co-infected patients: a cohort study

**DOI:** 10.1186/s12879-017-2196-0

**Published:** 2017-01-17

**Authors:** Valérie Martel-Laferrière, Roy Nitulescu, Joseph Cox, Curtis Cooper, Mark Tyndall, Danielle Rouleau, Sharon Walmsley, Leo Wong, Marina B. Klein, for the Canadian Co-infection Cohort Study Investigators, Lisa Barrett, Jeff Cohen, Brian Conway, Curtis Cooper, Pierre Côté, Joseph Cox, John Gill, Shariq Haider, Mark Hull, Marina Klein, Julio Montaner, Erica Moodie, Neora Pick, Anita Rachlis, Danielle Rouleau, Roger Sandre, Mark Tyndall, Steve Sanche, Marie-Louise Vachon, Sharon Walmsley, Alex Wong, David Wong

**Affiliations:** 1 Centre de Recherche du Centre hospitalier de l’Université de Montréal, 900 Saint-Denis, Montréal, Quebec H2X 0A9 Canada; 2 McGill University Health Centre, 1001 Decarie Blvd, Montreal, Quebec H4A 3J1 Canada; 3 Ottawa Hospital Research Institute, 501 Smyth Rd, Ottawa, Ontario K1H 8L6 Canada; 4 University of British Columbia, 2775 Laurel Street, 10th Floor, Vancouver, British Columbia V5Z 1M9 Canada; 5 B.C. Centre for Disease Control (BCCDC), 655 W 12th Ave, Vancouver, BC V5Z 4R4 Canada; 6 University Health Network, 101 College, Toronto, Ontario M5G 1L7 Canada

**Keywords:** HIV, Cocaine, Liver fibrosis, APRI score

## Abstract

**Background:**

Cocaine and crack use has been associated with HIV and HCV infections, but its consequences on HCV progression have not been well established. We analyzed the impact of cocaine/crack use on liver fibrosis progression in a cohort of HIV-HCV co-infected patients.

**Methods:**

A Canadian multicenter prospective cohort study followed 1238 HIV-HCV co-infected persons every 6 months between 2003 and 2013. Data were analyzed from 573 patients with positive HCV RNA, not on HCV treatment, without significant liver fibrosis (AST-to-Platelet Ratio Index (APRI) <1.5) or history of end-stage liver disease at baseline, and having at least two study visits. Recent cocaine/crack use was defined as use within 6 months of cohort entry. Incidence rates of progression to significant fibrosis (APRI ≥ 1.5) were determined according to recent cocaine/crack use. Cox Proportional Hazards models were used to assess the association between time-updated cocaine/crack use and progression to APRI ≥ 1.5 adjusting for age, sex, HCV duration, baseline ln(APRI), and time-updated alcohol abuse, history of other drug use and CD4+ cell count.

**Results:**

At baseline, 211 persons (37%) were recent cocaine/crack users and 501 (87%) ever used cocaine/crack. Recent users did not differ from non-recent users on gender, age, and CD4+ T-cell count. Over 1599 person-years of follow up (522 PY in recent users, 887 PY in previous users and 190 PY in never users),158 (28%) persons developed significant fibrosis (9.9/100 PY; 95% CI, 8.3–11.4); 56 (27%) recent users (10.7/100 PY; 7.9–13.5), 81 (28%) previous users (9.1/100 PY; 7.1–11.1), and 21 (29%) never users (11.1/100 PY; 6.3–15.8). There was no association between ever having used or time-updated cocaine/crack use and progression to APRI ≥ 1.5 (adjusted HR (95%CI): 0.96 (0.58, 1.57) and 0.88;(0.63–1.25), respectively).

**Conclusions:**

We could not find evidence that cocaine/crack use is associated with progression to advanced liver fibrosis in our prospective study of HIV-HCV co-infected patients.

## Background

Worldwide, the number of cocaine/crack users is estimated at 14–21 million people, including 5.7 million North Americans [1]. Cocaine/crack addiction is a significant problem in Canada. In the Canadian Alcohol and Drug Use Monitoring Survey, cocaine use was reported by 0.9% of persons surveyed in 2011 [2]. In the I-track study published in 2010, cocaine was the most frequently injected drug, with 52% percent of injection drug users (IDU) reporting having used it in the last 6 months [3]. Similarly, in the SurvUDI network study (2003–2008), 86% of IDU reported injecting cocaine and 68% reported smoking crack/freebase [4].

Cocaine/crack use has been associated with an increased risk of HIV and hepatitis C (HCV) transmission, even when the drug is not injected [5–7]. This phenomenon could result from mucous membrane lesions, sharing of material and at-risk sexual behaviours [8]. On the other hand, the impact of cocaine/crack on the natural history and treatment of HIV and HCV is an understudied phenomenon. This situation is due, in part, to the absence of a substitute for cocaine as compared to methadone and buprenorphine for opiates [9]. Nevertheless, it is plausible that cocaine/crack could accelerate liver fibrosis progression through vascular damage and recurrent episodes of ischemic acute hepatitis.

The aim of our study is to determine if cocaine/crack use is associated with accelerated liver fibrosis progression among HIV/HCV co-infected patients as measured by the APRI score.

## Methods

### Participants

The Canadian Co-Infection Cohort is an open prospective multi-centre cohort study of HIV/HCV co-infected individuals (*n* = 1238) recruited from 18 centers across Canada since 2003. It represents approximately 20% of the co-infected population under care in Canada. Inclusion criteria are: ≥16 year old, documented HIV infection by serological assay confirmed by Western blot, HCV infection documented by serological assay or, in case of false-negative serology, a positive HCV RNA. Socio-demographic and behavioral data are collected by self-reported questionnaires. Medical charts are reviewed for medical treatments and diagnoses and samples are collected at baseline and every 6 months. Details of the cohort can be found elsewhere [10].

Inclusion criteria for this analysis included participants with chronic HCV infection (positive HCV RNA), not receiving HCV treatment at baseline, who did not have significant liver fibrosis (see below) or a history of end-stage liver disease at baseline and who had at least two study visits. Patients were followed until their first outcome occurrence, initiation of HCV treatment or last study visit before database closure in December 2013.

### Cocaine/crack use

The exposure of interest was cocaine/crack use, either recent (within 6 months of a study visit), previous but not within 6 months, or never. Data concerning drug use were collected at every study visit. Questions evaluated types of drug used and routes of administration (injection vs. other) ever and in the last 6 months.

### Outcome: significant liver fibrosis

The AST-to-Platelet Ratio (APRI; (AST/ULN)/platelets × 100) is a non-invasive scoring system allowing for categorization of patients as free of significant fibrosis (score ≤ 0.5), with advanced fibrosis (≥1.5) or with cirrhosis (≥2). The main outcome of interest was occurrence of significant liver fibrosis defined as an APRI ≥ 1.5 [11].

### Statistical analyses

Descriptive statistics were used to characterize variables at baseline. Medians and interquartile ranges or numbers and proportions are reported for continuous and discrete variables respectively.

The following covariates of interest were measured: age, sex, ethnicity, income, body mass index (BMI), diabetes (determined based on glucose and fasting status), duration of HCV infection and time since HIV diagnosis and time updated HIV viral load and CD4+ cell count, antiretroviral therapy use, IDU, cocaine, crack and alcohol abuse in the previous 6 months. HCV duration was determined as time since first HCV diagnosis test, probable HCV infection according to patient, or first injection drug use, whichever was greater. Alcohol abuse was defined as more than six drinks at least once a month or more than two drinks on a typical day when drinking.

Incidence rates of progression to significant fibrosis (APRI ≥ 1.5) from baseline (cohort entry) were determined according to recent cocaine/crack use. Cox Proportional Hazards models were used to assess the association between time-updated recent cocaine/crack use and progression to APRI ≥ 1.5, while adjusting for baseline age, sex, HCV duration, ln(APRI) and previous use of cocaine/crack at at any time prior the baseline visit and time-updated current alcohol abuse, use of other drugs, and CD4+ cell count. The final model was constructed using covariates selected *a priori* as likely to be potential confounders. A sensitivity analysis was also performed by replicating the primary analysis using an APRI cut-off of 2 (cirrhosis) rather than 1.5. In case of missing variables, imputation rules were used. Indicator variables were imputed as false, continuous variables were imputed with their mean or median, depending on the shape of their distributions. All analyses were conducted using R version 3.3.0 (R Core Team, 2016).

## Results

**Fig. 1:**
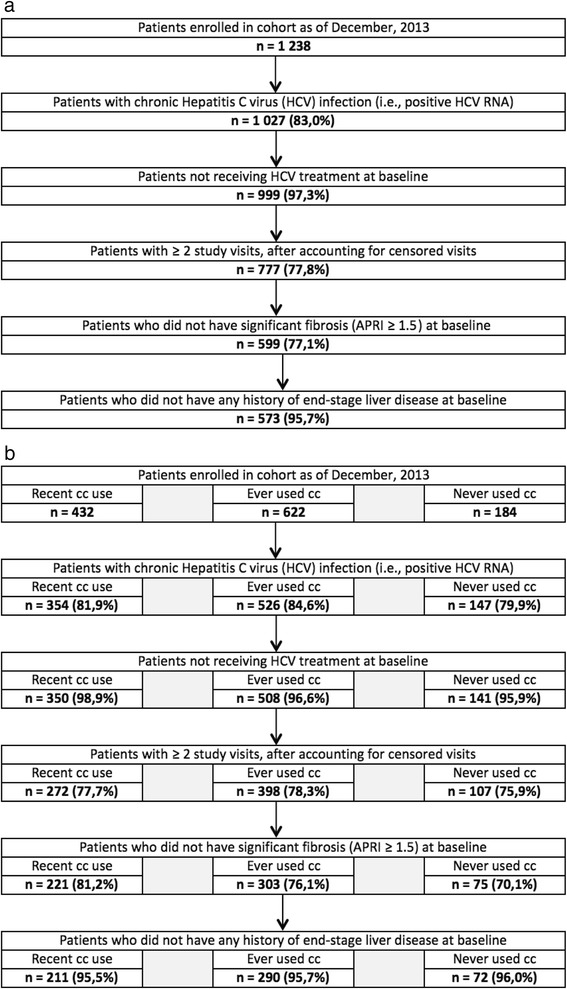
**a** Sample Selection Flow Chart. **b** Sample Selection Flow Chart Stratified by Group Legend: cc: Cocaine/crack

A total of 573 patients met inclusion criteria of whom, at baseline, 211 persons (36.8%) were recent cocaine/crack users, 290 (50.6%) persons previously used cocaine/crack but were not currently using, and 72 (12.6%) persons never used cocaine/crack (Fig. 1 and Table 1). Over the course of follow up, 47% of previous users and 3% of never users became recent users, while 79% of recent users remained recent users.

**Table 1:** Baseline characteristics of study population

	Total (*N* = 573)	Recent cocaine/crack use (*N* = 211)	Previously used cocaine/crack; not recent (*N* = 290)	Never used cocaine/crack (*N* = 72)
Follow-up time (years)^a^	2.3 (1.0, 4.1)	2.1 (1.0, 3.9)	2.4 (1.1, 4.5)	2.0 (0.8, 4.4)
Age (years)	44 (38, 49)	43 (38, 49)	44 (38, 48)	47 (40, 53)
Female	173 (30%)	69 (33%)	81 (28%)	23 (32%)
Aboriginal	86 (15%)	53 (25%)	31 (11%)	2 (3%)
Time since HIV diagnosis (years)	10 (5, 16)	10 (5, 14)	10 (6, 16)	13 (5, 20)
Duration HCV infection (years)	18 (10, 25)	19 (12, 26)	18 (11, 25)	10 (4, 20)
CD4 cell count (cells/μL)	389 (253, 550)	360 (220, 528)	390 (260, 550)	439 (300, 601)
HIV RNA load ≤ 50 copies/mL	340 (59%)	117 (55%)	175 (60%)	48 (67%)
Time since first start of ART (years)	6 (2, 11)	6 (2, 10)	6 (3, 11)	9 (2, 12)
cART regimen	453 (79%)	164 (78%)	227 (78%)	62 (86%)
Prior AIDS diagnosis	162 (28%)	65 (31%)	77 (27%)	20 (28%)
APRI	0.5 (0.4, 0.8)	0.5 (0.4, 0.7)	0.6 (0.4, 0.8)	0.6 (0.4, 1.0)
HCV RNA (log_10_ UI/mL)^b^	6.2 (5.4, 6.7)	6.1 (5.1, 6.6)	6.2 (5.5, 6.8)	6.2 (5.6, 6.7)
HCV treatment naïve	512 (89%)	197 (93%)	258 (89%)	57 (79%)
Alcohol abuse^c^	83 (14%)	41 (19%)	37 (13%)	5 (7%)

^a^: Median (IQR) or Number (%)

^b^: For HCV RNA only 307 (86/211 (41%) recent cocaine/crack users, 170/290 (59%) previous/not recent cocaine/crack users, and 51/72 (71%) non cocaine/crack users) had available quantitative HCV RNA values

^c^: Defined as >6 drinks at least once a month and >2 drinks on a typical day when drinking

Recent cocaine/crack users did not differ from previous users and never users on gender, age, and time since HIV diagnosis. At baseline, recent users were more likely to be be younger and of of Aboriginal origin, had lower CD4+ T-cells, were more likely to abuse alcohol, had longer median durations of HCV infection, but had lower median APRI scores and were more likely to be HCV treatment naive. The three groups did not differ with respect to reasons for censoring except that study withdrawal and HCV treatment initiations were more frequent among never users (Table 2).

**Table 2:** Reasons for censoring

	Recent cocaine/crack use	Previously used cocaine/crack	Never used cocaine/crack
	*n* = 211	*n* = 290	*n* = 72
Outcome (APRI ≥ 1.5)	56 (27%)	81 (28%)	21 (29%)
End of study period	99 (47%)	96 (33%)	21 (29%)
HCV Treatment initiation	15 (7%)	37 (13%)	13 (18%)
Death	11 (5%)	24 (8%)	2 (3%)
Lost to follow-up	23 (11%)	33 (11%)	7 (10%)
Withdrawal	7 (3%)	19 (7%)	8 (11%)

Over 1599 person-years of follow up (522 PY in recent cocaine/crack users, 887 PY in previous users, and 190 PY in never users), 158 (28%) persons developed significant fibrosis (9.9/100 PY; 95% CI, 8.3–11.4), of whom 56 (27%) were recent users (10.7/100 PY; 7.9–13.5), 81 (28%) were previous users (9.1/100 PY; 7.1–11.1), and 21 (29%) were never users (11.1/100 PY; 6.3–15.8).

Because drug use can change over time, a model was built with time-updated status for recent cocaine/crack use and recent other drug use (Table 3). Time-updated recent cocaine/crack use and ever using cocaine/crack at baseline were not associated with progression to an APRI ≥ 1.5 (recent: 0.88 (0.63–1.25) and ever (0.96 (0.58–1.57)). Female sex, higher baseline ln(APRI), current alcohol abuse, and lower CD4 counts were significantly associated with the fibrosis progression.

**Table 3:** Multivariate analysis of cocaine/crack use and liver fibrosis progression

	Recent cocaine/crack use	
	HR	95% CI
Time independent variables (at baseline)		
Cocaine/crack use ever at baseline	0.96	0.58, 1.57
Baseline age (per 10 years)	1.04	0.86, 1.27
Female sex	1.45	1.04, 2.01
Baseline HCV infection duration (per 5 years)	1.00	0.92, 1.10
Baseline ln (APRI)	2.84	1.94, 4.16
Time updated variables		
Recent cocaine/crack use	0.88	0.63, 1.25
Recent other drug use^a^	1.07	0.77, 1.49
Current alcohol abuse^b^	1.63	1.09, 2.42
CD4 count (per 100 cells/μL)	0.90	0.84, 0.96

^a^Heroin and opiates, steroids, methylphenidate, hallucinogens (PCP, LSD), benzodiazepine, barbiturates, methamphetamine, amphetamine, other

^b^Defined as >6 drinks at least once a month and >2 drinks on a typical day when drinking

Similarly, cocaine/crack use was not associated with progression to an APRI ≥ 2 in both time-updated recent users and previous users at baseline (recent: 0.69 (0.45–1.04) and ever: 0.83 (0.50–1.40)) (Table 4). Female sex, higher baseline ln(APRI score), current alcohol abuse, and lower CD4 count were also associated with achieving and APRI ≥ 2.

**Table 4:** Multivariate analysis of cocaine/crack use and liver fibrosis progression using cut-off of 2 for APRI

	Recent cocaine/crack use	
	HR	95% CI
Time independent variables (at baseline)		
Cocaine/crack use ever at baseline	0.83	0.50, 1.40
Baseline age (per 10 years)	1.04	0.83, 1.29
Female sex	1.48	1.03, 2.13
Baseline HCV infection duration (per 5 years)	1.05	0.95, 1.16
Baseline ln (APRI)	2.54	1.69, 3.81
Time updated variables		
Recent cocaine/crack use	0.69	0.45, 1.04
Recent other drug use^a^	1.19	0.82, 1.72
Current alcohol abuse^b^	1.76	1.14, 2.72
CD4 count (per 100 cells/μL)	0.91	0.85, 0.98

^a^Heroin and opiates, steroids, methylphenidate, hallucinogens (PCP, LSD), benzodiazepine, barbiturates, methamphetamine, amphetamine, other

^b^Defined as >6 drinks at least once a month and >2 drinks on a typical day when drinking

## Discussion

In this longitudinal study of liver fibrosis progression in HIV-HCV co-infected patients, we were unable to demonstrate an association between cocaine/crack use and evolution of liver fibrosis as measured by APRI score. Not surprisingly, alcohol abuse, CD4 cell count and baseline APRI score were predictors of progression to advanced fibrosis. The association between female sex and liver fibrosis progression although not generally seen in HCV mono-infection was previously noted in the Canadian Co-infection cohort and other co-infected studies [12, 13].

The influence of cocaine on liver pathology has been studied in animal models. Cocaine has been associated with parenchymal necrosis and microvesicular changes in hepatic architecture [14]. Cocaine is metabolised in the liver by CYP450 to norcocaine [15]. Norcocaine is then transformed into free radicals which cause liver damage [14]. Decreased ATPase activity in isolated rat liver mitochondria was also reported [16]. In addition, liver damage can result from damage to other organs through hyperpyrexia, hypotension and rhabdomyolysis [15, 17].

With respect to fibrosis and liver function, data from clinical human studies are currently limited. In a retrospective study, transplant recipients who received a liver from a donor who was a cocaine user had higher levels of prothrombin time and AST on post-operative day one and more graft loss within 3 months as compared to recipients of livers from non-cocaine users [14]. There was also more primary non-function and lower graft survival in the cocaine group. One potential confounder in this study was that cocaine users were also more likely to drink alcohol. Nevertheless, the study suggests at least a subclinical impact of cocaine on liver function [14].

On the other hand, Foucher et al. study contradicts the hypothesis of accelerated liver disease progression among cocaine users [18]. Cocaine-snorting was not associated with elevated transient elastography scores in their study performed on 298 drug users. However, those who never snorted cocaine and had less than 21 drinks per week were more likely to be HCV seropositive raising concerns about the generalizability of these findings [18]. Multiple other studies have shown a high correlation between cocaine use and HCV infection, [19, 20] although none has investigated the effect of cocaine on liver disease progression.

Our study is the first to evaluate systematically the presence of an association between cocaine/crack use and liver fibrosis. This was done in a large prospective cohort of HIV-HCV co-infected patients, representative of the population of co-infected patients in care in Canada. We controlled for major known confounders that could be linked with cocaine use and liver disease risk.

Among study limitations, the most important is that data about quantity and route of administration other than injection are not collected in our study questionnaire. Consequently, we cannot exclude a dose-related effect of cocaine/crack on liver fibrosis. Concerning route of administration, we chose to combine all routes of administration together based on previous studies showing that similar serum concentrations are achieved when these drugs are injected and smoked although some studies suggest snorted cocaine leads to lower plasma drug concentration [21]. Because almost the entire patient population (87%) had used cocaine or crack at some time, focusing only on recent use could miss potential long-term effects of cocaine on the liver. In order to account for consumption changes over time, we used a time-updated variable for recent cocaine/crack use. However, we did not have a measure of duration or intensity of such drug use prior to cohort entry.

Arguably APRI score is an imperfect surrogate for liver fibrosis and is not the gold standard for liver fibrosis evaluation. Nevertheless, because of cost and potential safety risks, it is not possible to perform repetitive liver biopsy in a cohort study. In addition, even if transient elastography is now considered as a validated non-invasive test for liver fibrosis and is well suited for repeated testing, it was not available when the cohort was formed and is still not available to all our study sites. APRI is widely used in clinical practice and has been recommended by the World Health Organization [22]. Despite a meta-analysis showing a trend toward less accurate results for APRI in co-infected than mono-infected patients, this difference was not significant in meta-regression analysis and APRI has been validated in our HIV/HCV co-infected patients [11, 23]. Multiple studies have used the APRI score and demonstrated its value in predicting liver fibrosis progression, hepatic decompensation and death [11, 24–27]. The APRI score has been shown to perform similarly to other markers of liver fibrosis including FIB-4 [28]. The APRI score lacks sensitivity and may misclassify individuals as not having fibrosis, however is highly specific for fibrosis stage ≥ F2 (0.93, 95% CI: 0.91, 0.94) [23]. Thus using APRI ≥1.5 as an outcome is conservative. While we may have underestimated the degree of fibrosis present among those with scores below 1.5, any such under-ascertainment is unlikely to vary by exposure to cocaine. Nevertheless, because there is some controversy concerning the clinical significance of progression to an APRI ≥ 1.5, we conducted a sensitivity analysis with an APRI cut-off value of 2, corresponding to liver cirrhosis. Similar to the other models, recent cocaine/crack use or ever using cocaine/crack was not associated with APRI progression.

## Conclusions

In conclusion, while cocaine/crack use is certainly associated with high-risk behaviours related to HCV infection and to potential acute liver injury, we were unable to demonstrate an association between these drugs and chronic liver fibrosis progression.

## Abbreviations

APRI: AST-to-Platelet Ratio
BMI: Body mass index
HCV: Hepatitis C
IDU: Injection drug users
PY: Person-years
